# A density estimation model of plateau pika (*Ochotona curzoniae*) supporting camera‐monitoring programs

**DOI:** 10.1002/ece3.7865

**Published:** 2021-07-13

**Authors:** Ying‐Hui Jia, Jun Qiu, Cang Ma, Jin‐Zhao Wang, Guang‐Qian Wang, Fang‐Fang Li

**Affiliations:** ^1^ College of Water Resources & Civil Engineering China Agricultural University Beijing China; ^2^ State Key Laboratory of Plateau Ecology and Agriculture Qinghai University Xining China; ^3^ State Key Laboratory of Hydroscience & Engineering Tsinghua University Beijing China

**Keywords:** activity intensity, burrow system, camera trapping, density estimation model, Monte Carlo method, plateau pika, population density

## Abstract

As an important species in the Qinghai‐Tibet Plateau, the roles played by plateau pikas in grassland degradation and protection are controversial. The behavior characteristics and population density of this species are important in answering this question, but these traits have not been fully elucidated to date. Camera‐capture methods have been used widely in recent years to characterize or calculate population density with the advantage of simple operation and nonintrusive investigation. However, establishing the relationship between actual population density and monitoring data with the condition that individual identification is not possible is a major challenge for this method. In this study, a model composed of a behavioral module and a burrow system module is proposed and applied to simulate the moving path of each individual pika. Based on Monte Carlo method, the model is used to develop the relationship between population density and recorded capture number, which is compared with the results derived from the random encounter model (REM) based on field observations. The simulated results mixed with the calculated density based on observation data could reach *R* ^2^ = 0.98 using linear fitting, with proper parameter settings. A novel index named activity intensity of pikas per population density is also proposed, providing information on both the ecological physical characteristics and monitoring space. The influence of different parameters on this index, mainly the pika number per burrow system, pika activity time outside the burrow, and activity intensity, is discussed. The proposed methodology can be applied to different scenarios in further studies when behavioral characteristics of pikas change for such reasons as climate change and vegetation degradation.

## Graphical abstract

A digital twin model of pikas’ activity composed of a behavior model and a cave system model is proposed. The model is used to estimate the population density of pikas, compared with the calculated density derived from random encounter model (REM) and field observation. A novel index named activity intensity of pikas per population density is also proposed, indicating the information of both the ecological physical parameters and the monitoring space.

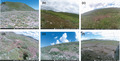

## 1 INTRODUCTION

The Qinghai‐Tibet Plateau is the largest plateau in China and the highest plateau in the world, known as the “Roof of the World.” The meadow on the Qinghai‐Tibet Plateau are an important green barrier maintaining the ecological balance of the region, and meadows are also one of China's important animal husbandry bases. In recent years, grassland degradation on the Qinghai‐Tibet Plateau has attracted increasing attention from scholars. Liu et al. (2008) reported that more than 36% of the grassland on the Qinghai‐Tibet Plateau has been degraded, and the area of moderately and severely degraded grassland accounts for 12% of the total land area according to Chinese national standards, although various versions of evaluation criteria exist (Harris, 2010).

Plateau pikas (*Ochotona curzoniae*) are endemic to the Qinghai‐Tibet Plateau and are small nonhibernating herbivorous mammals. They are large in number and tend to gather in space (Smith & Gao, 1991). They live on slopes and river valleys where the soil is relatively loose, and each pika family has a burrow system with multiple entrances and exits (Schaller, 1998). The burrow system, which composed of hidden burrows and habitat burrows, is intricate and distributed radially with nests as the center (Pan & Wangdui, 2016). Grassland degradation on the Qinghai‐Tibet Plateau in recent years has triggered a heated discussion regarding pikas in academic circles: Is the plateau pika a pest or is it a key species that maintains the in situ ecological stability and biodiversity (Smith, 1999)? Although it is rare today to find literature wholeheartedly supporting the idea that pikas are responsible for grassland degradation, some studies do reveal pika‐related environmental degradation. When the density of pika burrows exceeds a certain critical value, grassland biomass generally decreases with the increasing density of pika burrows, which indicates that the extensive existence of pikas is not conducive to grasslands (Liu et al., 2017). Aboveground biomass and plant height show a significant negative exponential trend with increasing pika burrow density, while the population density is demonstrated through field trials to be related to the grazing system and stocking rate (Wang et al., 2020). However, a grazing experiment conducted by Pech et al. (2007) showed that there is no evidence that forage production increases in areas where the number of pikas is controlled or that controlling pikas improves the lives of Tibetan herders. Some studies have found that pika eradication campaign could reduce the infiltration rate of water (Wilson & Smith, 2015), decrease the abundance of carnivores (Palden et al., 2016), and cause the loss of avian species richness (Lai & Smith, 2003), indicating the important functional ecological traits of pikas (Smith et al., 2019). Small mammalian herbivores such as pikas serve as mediators of plant community dynamics (Bagchi et al., 2006). Zhao et al. (2020) claimed that the existence of pikas is vital to maintaining the diversity of plateau plants and the survival of a variety of predators. In addition, the burrowing behavior of pikas increases soil permeability (Zhao et al., 2020). Therefore, plateau pikas are also considered water conservancy engineers on the plateau. Harris (2010) summarized and analyzed the causes of grassland degradation on the Qinghai‐Tibet Plateau from past studies, and he believed that the increase in pikas has less impact on pastures and that such phenomenon is more suitable as an indicator of grassland degradation. Nevertheless, according to more recent research, a different opinion has been put forward illustrating that whether plateau pikas are harmful to grassland depends on population density (Sun et al., 2015; Wei, Hi, & Zheng, 2020). Thus, the population of this species is meaningful not only to its own development but also to the sustainability of the local ecosystem.

Knowledge and understanding of the pika's living environment (burrow structures, light conditions, climate, surrounding vegetation types, and coverage) and habits help improve our knowledge of their role in grassland processes such as degradation. For example, pika food preferences can help scientists better predict the growth of certain vegetation. By determining the extent and intensity of pika's burrowing behavior, one can obtain a quantitative or qualitative estimation of its influence on soil permeability. Various studies related to pika have been published recently (Qu et al., 2017; Tang et al., 2019; Wei et al., 2013; Zong & Wuping, 1987). The density and survival of plateau pikas experience seasonal fluctuations, while demographic features and climate together regulate the population dynamics (Qu et al., 2017). Observations show that the length of time that plateau pikas are active mainly depends on the length of daylight and that the beginning and end time of their ground activity outside burrows is directly controlled by light intensity (Zong & Wuping, 1987). The seasonal variation in the plateau pikas’ activity time is basically in line with seasonal sunlight variation. When the daytime is long, the pika's activity time is long, otherwise it is short; in severe weather, such as strong wind, heavy snow, heavy rain, their activity is obviously reduced or even halted. Smith and Gao (1991) reported that pikas are social animals with a population density as high as 300/hm^2^. Wei et al. (2013) found that pikas’ burrow systems are very complex and related to environmental conditions such as orientation and temperature. It is claimed that a smaller entrance slope has a greater probability of facing sunlight; and there exists a significant temperature difference among burrow entrances facing different directions. All information indicates that plateau pikas take the temperature, airflow convection, and resistance to cold into account when digging burrows. Tang et al. (2019) studied the spatial distribution of pika burrows by analyzing images taken by drones; the results showed that the size of the burrow entrances is approximately 0.01 m^2^ and the number of burrow entrances has an obvious convex quadratic relationship with vegetation coverage. Moreover, the influence of the burrow on the surrounding grassland is mainly focused within 20 cm of the burrow. Wei and Zhang (2018) made a detailed study on the pika burrow system, which showed the possibility of a temporary burrow transforming to a permanent one.

Generally, current research on plateau pikas’ behavior is mostly based on limited observations, and there is a lack of further detailed research on specific behavioral patterns and group behavior rules, especially with quantitative analysis. Although Smith et al. (1986) made a detailed classification of pikas’ behavioral patterns, mainly divided into social behaviors (including chasing, playing, following, and tidying up hair) and nonsocial behaviors (including eating, vigilance, digging, and resting), the duration, frequency, and displacement of various behavior patterns need to be further quantified.

The population density of pikas is an important index in evaluating their ecological function (Smith et al., 2019). The traditional methods of pika population density investigation mainly include the quadrat sampling method (Lamontagne & Joseph, 1993), mark recapture (Roy et al., 2014), and removal sampling (Schmidt et al., 2015), which require complex artificial means to capture individuals in the population, such as bait, plate clamp, and burrow blocking. To avoid complicated investigation, some studies have applied the number of live burrows to characterize population density (Wu et al., 2019), such as the trace line method based on feces and the use of burrows. Wei, He, Zheng, He, et al. (2020) estimated absolute density using the removal sampling method and compared it with the total burrow density (TBD), active burrow density (ABD), and direct counting density (DCD), and their results reveal that neither TBD nor ABD reflect the true absolute pika density, while DCD may be a powerful alternative. However, the reliability and accuracy of DCD depend greatly on the observer and survey time, and it is unlikely to conduct long time continuous observation using this method. Moreover, DCD could not conduct calibration afterward, especially in the case when similar small animals are present in the habitat. Chen et al. (2008) measured the number of living burrows by plugging burrows in addition to a method of directly capturing pikas. Qu et al. (2011) demonstrated that the density obtained by the walked transects method is lower than that of the mark–recapture method, and it is recommended as an index of relative density in large‐scale assessments in alpine grassland. In addition to high consumption, the population density determination methods above either artificially interfere with the pikas’ habitat or lack accuracy. With the rise of animal protection and the idea of reducing habitat interference, noninvasive, efficient, and convenient camera‐trapping technology has been widely applied. This technology uses an infrared trigger automatic digital camera to capture species and estimates the number of populations based on the captured‐image information (Karanth et al., 2006; Ma et al., 2011). However, it is difficult and controversial to link population density with actual monitoring data, and the accuracy of population density estimation needs to be further improved (Burton et al., 2015).

Based on probability and statistical theory, Monte Carlo method uses random numbers or, more commonly pseudo‐random numbers to practical calculation problems for population. Its basic principle is to link the problem to be solved with a certain probability model and then conduct statistical simulation or sampling to obtain an approximate solution to the problem by computer. Monte Carlo method omits complicated mathematical derivation and calculation processes and has a wide range of applications in ecology. Giró et al. (1986) proposed a model describing the state of ecological populations changing with time under known environmental conditions and obtained simulation results consistent with theoretical research. Bonner and Schofield (2014) used the Monte Carlo method and mark–recapture method to calculate population size more efficiently and accurately. Avgar et al. (2013) established a behavior model that describes individual motion trajectories combining the cognitive process of individuals and environmental information, which can make probabilistic decisions based on efficiency and benefits. Their research verified the feasibility of using probabilistic models to describe the natural behavior of species. Song et al. (2015) also used the Monte Carlo method to assess the uncertainty of the weight of the ecological vulnerability index.

In this study, camera‐trapping technology was used for field observations of pikas on the Qinghai‐Tibet Plateau. In addition, a probability module describing the behavior of pikas based on the Monte Carlo method, as well as a pika burrow system module, was established to simulate individual behaviors in a pika population. Compared with traditional methods mentioned earlier which are based mainly on statistics and may severely deviate in some cases, the method proposed in this paper can be used to interpret or estimate the density of pikas from a physiological point of view. An index of the activity intensity of pikas per population density is proposed to be utilized as the standard for comparison between field observation and numerical simulations, which reflects monitoring spatial information and pika behavior information simultaneously. By changing the activity time of pikas outside the burrow, the maximum and minimum of the number of pikas in each burrow system, and the average speed of high‐ and low‐intensity activities of pikas, the simulated activity intensity of pikas per population density is obtained to describe the linear relationship between population density and capture number, reflecting different behavioral characteristics. The proposed model can be further applied to different scenarios when the behavioral characteristics of pikas change for certain reasons, such as climate change and vegetation degeneration.

## 2 STUDY AREA

**FIGURE 1:**
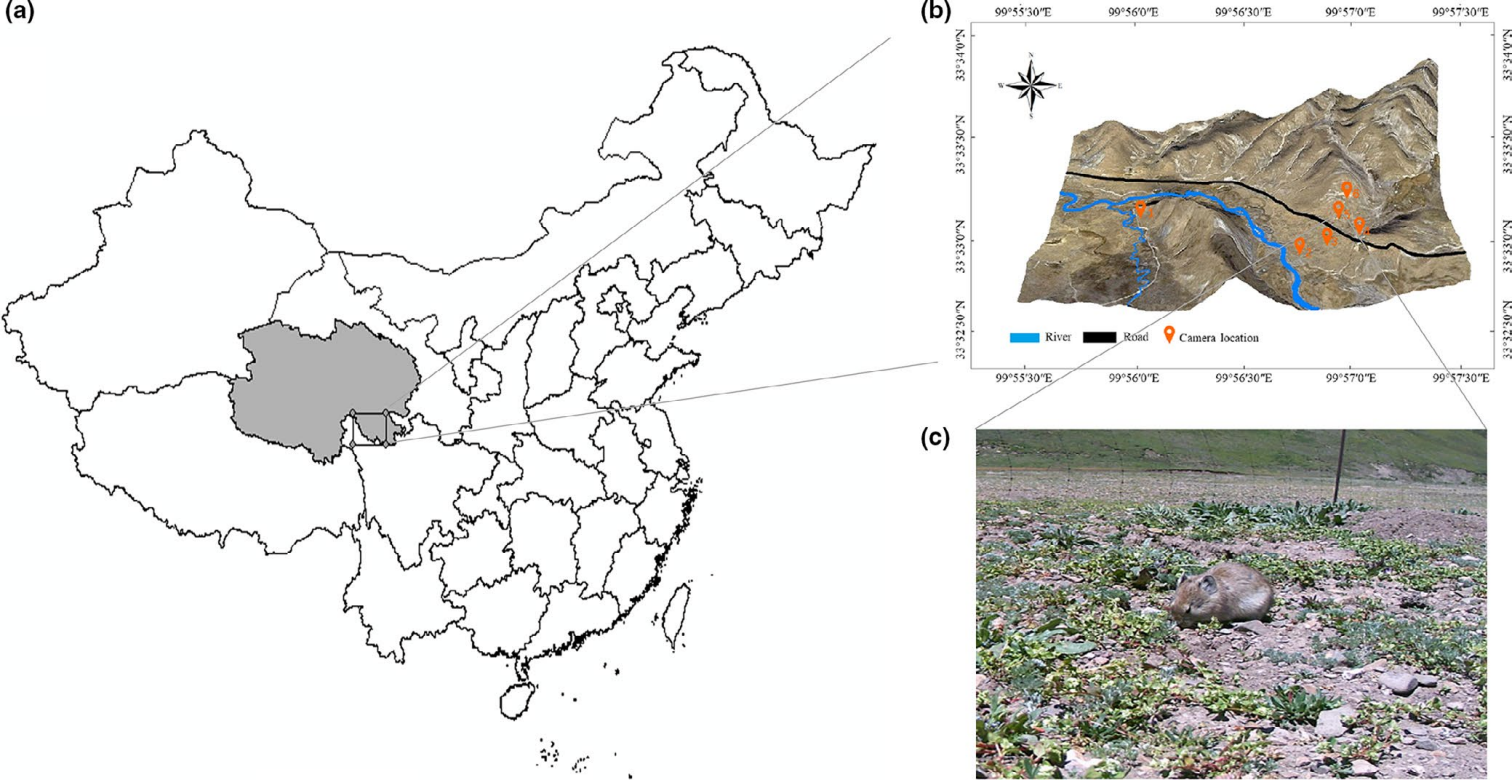
(a) Field observation area; (b) camera location point; (c) actual photograph of the plateau pika

Field observations were implemented in Dari County, which is located in the southeastern part of the Qinghai‐Tibet Plateau with geographic coordinates of 98°15′–100°33′ east longitude and 32°36′–34°15′ north latitude, as shown in Figure 1. The average altitude of Dari County is over 4,200 m. The county is subjected to an alpine and semi‐humid climate and is located in the “Sanjiangyuan National Nature Reserve” in China.

**FIGURE 2:**
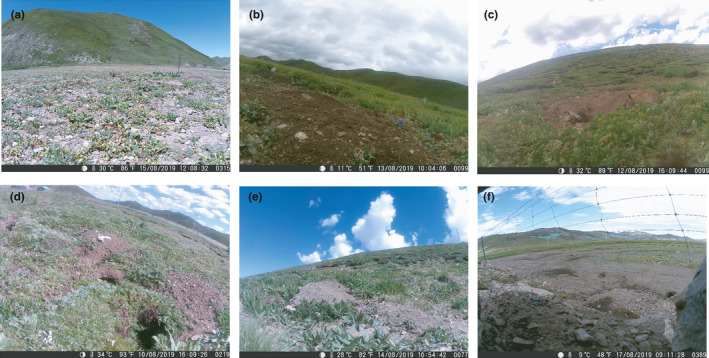
Vegetation conditions at different locations of cameras (a) 100 m from the riverbank; (b) 300 m from the riverbank; (c) gentle sunny slope; (d) on the side of the road; (e) steep sunny slope; (f) benchland of the river

Six cameras were installed at six different habitat locations: at a benchland of the Jiqu River, at 100 m and 300 m from the river bank of the Jiqu River, beside a road, on a gentle sunny slope, and on a steep sunny slope. Every camera was placed on the ground and had at least one pika burrow in sight. The vegetation condition in the six locations is not exactly the same, as shown in Figure 2. Seven‐day monitoring was conducted from 12 August to 18 August 2019. Due to unexpected natural conditions and camera malfunction, normal operation days varied for different cameras. The “foresafe H885” field infrared cameras were applied under the photo and video mode, and the recording time was set to be 30 s. The camera operated 24 hr a day. When an animal entered the monitoring range of the camera sensor, the camera would be triggered, a picture recording the shooting date and time would be taken, and then, a video 30 s in length would be recorded. Afterward, the camera would return to the standby mode. An actual photograph of a plateau pika is shown in Figure 1c. It was assumed that the population density varied with different locations.

## 3 METHODOLOGY

**FIGURE 3:**
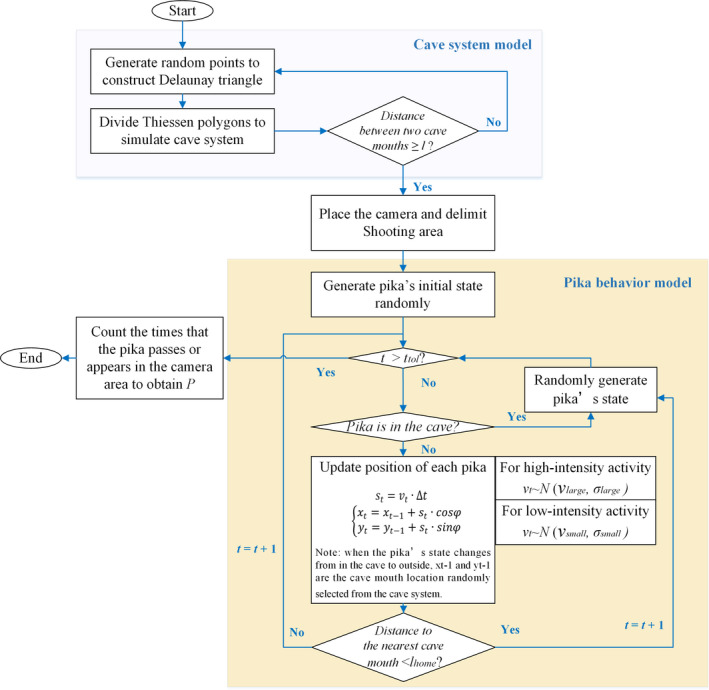
Overall flow chart of the simulation of group behavior of plateau pikas

The first step of this study was to set up an infrared camera in the field to obtain direct observation data of pika activity frequency. The data were then applied to calculate the density. Then, a model composed of burrow system module and a pika behavioral module was established to determine the relationship between density and capture number with proper parameter settings. The former module was developed to simulate the complex burrow structure and population distribution of pikas. The purpose of the latter module was to describe the movements of pikas. Figure 3 shows the flowchart of the proposed model with a detailed introduction to the two modules. During the process of model operation, burrow structure and population distribution were first generated by the burrow system module. Pikas then perform actions around their burrows, which was done by the pika behavioral module. Next, the results obtained through field observation were compared with those from simulations for validation. Finally, investigations into model structure and interactions between parameters are discussed in Section 4. Detailed information on the field observation and the modules is presented in the following sections.

### 3.1 Field observation

**FIGURE 4:**
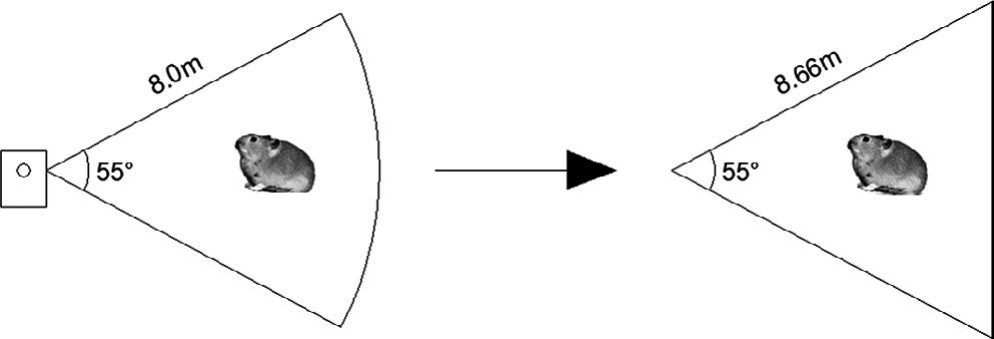
Camera range

Camera‐trapping technology has recently been widely used to obtain ecological data, such as the distribution, diversity, behavior, and structure of biotic populations (Linkie et al., 2013; Rovero & Marshall, 2009; Sollmann et al., 2013). In this study, according to geography, hydrology, traffic and other conditions of the study area, and the number of cameras and the camera‐shooting range, different areas were used. There were six cameras located at six different places, all placed on the ground operating for a week. The photograph and video modes were applied for each camera, that is, when a pika appeared or passed through the camera's shooting area, the camera was triggered to shoot and recorded for 30 min. The size of the camera's shooting area is one of the decisive factors for the capture number. The detection area of the camera used in the field was an arc‐shaped area with a central angle of 55° (0.96 mrad) and a detection radius of 8 m. During the simulation process, it was simplified to an equivalent isosceles triangle with the same area, as shown in Figure 4, with a side length of 8.66 m. While counting the pikas in the photographs taken by cameras, the corresponding 30‐s videos were also examined. When the interval between two successive shots was short and the behavior or position of the pika in the videos was consecutive, we assumed it was the same pika and counted it only once. This prevented multiple counts when pikas frequently visited the same entrance in a short period of time. The observation data were thought to be reliable since preparation work had been done for more than several weeks, and the pictures and videos were carefully checked and counted.

The population density *D* is calculated using the random encounter model (REM) by Rowcliffe et al. (2008) based on the principle of the gas molecule collision rate, which can estimate population density without identifying individuals. The population density derived from a single camera is as follows:

(1) $$D=\frac{y}{t}\frac{\pi }{vr(2+\theta )}$$

where *y* is the total number of independent photographs captured by the camera; *t* is the number of days the camera was deployed; *v* is the daily moving speed of the animal; *r* is the radius of the camera's detection area; and *θ* is the camera's detection angle. In this study, *r* = 8 m, and *θ* = 55° (0.96 rad). Zhang et al. (2013) set three values of *v* in REM model ($v_{1}=0.1\;\text{km}/d,\;v_{2}=0.2\;\text{km}/d,\;v_{3}=0.3\;\text{km}/d$) and derived three estimated population density of small rodents, which were compared with the population density *D* estimated by the marker recapture method. According to their study, the daily moving speed of pikas was set to be 0.3 km/day in this study.

The actual field shooting data and the estimated population density of pikas in different locations derived from the REM are shown in Table 1. The density of pikas was different in different regions. It was the highest in the area 100 m away from the river bank and lowest on the steep sunny slope.

**TABLE 1:** Estimated population density of pikas by the REM at different locations

Location	100 m from the river bank	300 m from the river bank	On the side of the road	Gentle sunny slope	Steep sunny slope	Benchland of the river
Normal working days	6	7	4	7	6	5
Number of independent photographs	271	270	130	201	105	161
Average daily number of triggered shootings	45	39	33	29	18	32
Estimated population density (${\text{ha}}^{‐1}$)	200	171	144	127	77	142

### 3.2 Activity intensity of pikas per population density *λ*

Under certain layout conditions, the frequency at which the infrared camera is triggered by pika activity was related to both the population density of the pikas and the activity intensity of the pika. For a given pika activity intensity, the camera is triggered more frequently by pikas with higher population density, while for a given pika population density, the camera is triggered more frequently by pikas with higher activity intensity. To describe such a relationship, the index of activity intensity per unit population density *λ* was proposed in this study, as defined in Equation (2), which was taken as the comparison standard between field observation and numerical simulations:

(2) λ=P/D(ha)

where *P* is the shooting frequency of the camera, and *D* is the population density of pikas calculated by Equation (1) from field observations. This parameter essentially indicates the information of both the monitoring space and pika behavior. For the proposed model, since the pika density was the same throughout the simulated area, we used the average number of shots for all days from all cameras to calculate *P*.

(3) $$P=\frac{\sum_{j=1}^{T}\sum_{i=1}^{N}q_{\mathrm{ij}}}{T}/N$$

where $q_{\mathrm{ij}}$ is the capture number taken by the *i*th camera on the *j*th day; *N* is the total number of cameras; and *T* is the total number of days of the observations. For field observations, the pika density of each camera set point is assumed to be different, and therefore, *P* represents the average capture number per day at each plot.

### 3.3 Pika burrow system module

**FIGURE 5:**
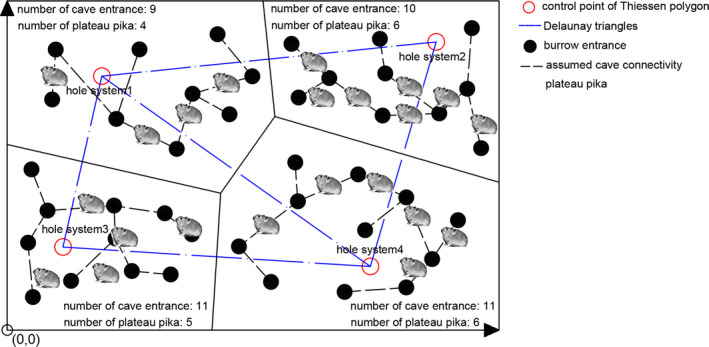
Schematic diagram of the burrow system structure and pika distribution

The pika burrow system module is developed to simulate the burrow structure and population distribution. The simulation range is a square with a side length of 1,000 m, in which 2,500 control points (represented by hollow red dots in Figure 5) are randomly generated. A Thiessen polygon (also called a Voronoi diagram) (Okabe et al., 1994) is a set of continuous polygons composed of vertical bisectors connecting two adjacent points. The distance from any point in a Thiessen polygon to the control points constituting the polygon is less than the distance to the control points of other polygons. In this study, Thiessen polygons were used to simulate the topotaxy of pika burrow system.

First, 2,500 control points were randomly generated in the simulation area. A Delaunay triangulation was constructed by connecting the control points while avoiding intersected lines. Thiessen polygons were divided according to the given boundary conditions, each of which represented the area occupied by a burrow system. These Delaunay control points can be estimated as the center of the pika burrow system. With the given maximum and minimum number of burrows in a burrow system, $H_{\max}$ and $H_{\min}$, respectively, as well as the minimum distance between any two burrows in the burrow system *l*, burrows in each burrow system were randomly generated. Then, a certain number of pikas were randomly generated for each burrow system given the maximum and minimum number of pikas for each burrow system $Q_{\max}$ and $Q_{\min}$, respectively. Since $Q_{\max}$ and $Q_{\min}$ directly determine the overall density of pikas in the simulation range, the variation in capture number with density was obtained by changing these parameters. To further illustrate their role in model performance, an influence study was conducted and is presented in results and discussion sections.

Learning from the characteristics of the Thiessen polygon, the distance from the burrow to its control point is less than that to the control points of other burrow systems. All pikas are assumed to be in the burrow at the initial stage. Figure 5 is a schematic diagram of the burrow system structure and pika distribution. It should be noted the dotted lines in Figure 5 only represent the assumed connectivity instead of the real path underground, which is unknown and difficult to describe. In the proposed methodology, only the information of the surface entrances is used to indicate the locations where pikas get in and out of the burrows, while the underground path of pikas is not tracked. Therefore, the structure of the underground part of burrows is not considered.

### 3.4 Pika behavior module

The pika behavior module is designed to simulate the ground movement of pikas. The pika's daily ground activity time is $t_{\text{tol}}$, and the fixed time length of each step when the pika goes out is Δt. As the actual camera recording time was 30 s, Δt was set to 30s to facilitate counting the capture number. Each pika has two alternative states at each time step, that is, outside or inside the burrow. In the burrow, a pika may store food, defecate, etc. When a pika is outside the burrow, it exits from a random burrow mouth in the burrow system to go out.

The behavior of pikas when going out is classified into two categories: high‐intensity activities, such as running; and low‐intensity activities, such as foraging and playing, as shown in Table 2. The average speed of high‐intensity and low‐intensity activity are set to be $v_{\text{large}}$ and $v_{\text{small}}$, respectively. In each time step, a random speed is generated for each pika according to Gaussian distribution, taking $v_{\text{large}}$ or $v_{\text{small}}$ as the mean value and $\sigma_{\text{large}}$ or $\sigma_{\text{small}}$ as the root mean square for high‐intensity and low‐intensity activity, respectively, as shown in Equation (4):

(4) $$v=\left\{\begin{array}{c} ∼N(v_{\text{large}},\sigma_{\text{large}})\;\text{for}\;\text{high - intensity}\;\text{activity,}\;\text{such}\;\text{as}\;\text{running}\\ ∼N(v_{\text{small}},\sigma_{\text{small}})\;\text{for}\;\text{low - intensity}\;\text{activity,}\;\text{such}\;\text{as}\;\text{foraging} \end{array}\right.$$

**TABLE 2:** Definition of activity state of pikas

Initial state	Inside burrow	Outside burrow	
		High‐intensity activity	Low‐intensity activity
Activity	Storage/breed/defecation/…	Running/chasing/…	Foraging/resting/alert/fighting/playing/ tidying up hair…
Velocity		$∼N(v_{\text{large}},\sigma_{\text{large}})$	$∼N\left(v_{\text{small}},\sigma_{\text{small}}\right)$

The position of the pika is updated at each step according to Equation (5):

(5) $$\left\{\begin{array}{c} x_{t}=x_{t‐1}+s_{t}\cdot \cos\varphi \\ y_{t}=y_{t‐1}+s_{t}\cdot \sin\varphi \end{array}\right.\;\varphi \in \left[0,2\pi \right]$$

where $x_{t}$ and $y_{t}$ are the coordinates of the pika's position after time step *t*, using the bottom left as the original point, as shown in Figure 5; $s_{t}$ is the distance the pika passed during the *t*th time step, and $s_{t}=v_{t}\cdot \Delta t$.

The moving direction of a pika at each step is random, that is, φ is random. When the pika is outside, it returns to the burrow mouth when its distance to the burrow entrance is less than a threshold $l_{\text{home}}$, and then, the pika's state becomes inside. Otherwise, the pika continues to act outside. A boundary condition is also examined, that is, when the pika exceeds a certain boundary during its movement, it immediately reenters the simulation area from the corresponding opposite side.

According to the above description, it is obvious that $t_{\text{tol}}$, $v_{\text{large}}$, and $v_{\text{small}}$ may have significant impact on the outside journey of pikas. As these parameters increase, intensified activity should be observed, leading to a higher probability of being filmed. To evaluate their impact, experiments were conducted to see how the model results varied with different parameter settings.

### 3.5 Implementation and validation

The overall flow chart of the simulation is shown in Figure 3. First, 2,500 control points were generated randomly to divide Thiessen polygons in a range of 1,000 m × 1,000 m, and each Thiessen polygon represented a burrow system. A certain number of burrows in each burrow system was also generated randomly given the minimal and maximal number of burrows, and a certain number of pikas were allocated to each burrow system within the given range of numbers. Then, the activities of all pikas on each day were simulated by the pika behavioral module. When the total activity time of a certain pika on the ground reaches $t_{\text{tol}}$, it stops moving on the current day. The initial state of all pikas on each day was set to be in the burrow. When a pika appears or passes the camera‐monitoring range during its ground activity, the camera shoots once. The parameters used in the simulation and the initial values are listed in Table 3.

**TABLE 3:** Parameters used in the simulation and the corresponding initial values

Parameters	Value	Reference
The length of simulation area (*L*)	1,000 m	
Number of burrow system (*n*)	2,500	
Ground activity time of pika ($t_{\text{tol}}$)	20 min	
Total simulation days ($D_{\text{tol}}$)	4 days	
The minimum distance between two burrows in the same burrow system (*l*)	2 m	
The maximum number of pika burrows for each burrow system ($H_{\max}$)	11	Shi (1983)
The minimum number of pika burrows for each burrow system ($H_{\min}$)	9	
The maximum number of pika for each burrow system ($Q_{\max}$)	6	Smith et al. (1986), Liang (1981)
The minimum number of pika for each burrow system ($Q_{\min}$)	4	
Time step (Δt)	0.5 min	
The maximum distance traveled per time step ($d_{\max}$)	30m	
The speed of motion during vigorous activity ($v_{\text{large}}$)	20 m/min	
The speed of movement during low‐intensity activities ($v_{\text{small}}$)	2 m/min	Wang et al. (2005)
Root‐mean‐square velocity during vigorous motion ($\sigma_{\text{large}}$)	2 m/min	
Root‐mean‐square velocity during low‐intensity motion ($\sigma_{\text{small}}$)	0.2 m/min	
The critical distance for pika returning to the burrow ($l_{\text{home}}$)	1 m	

There are a total of six camera‐shooting areas in the simulation. It is important to note that since population density is set to be consistent throughout the entire region in the model, only one density can be given at a time. However, in actual field experiments, we believe that the density of pika varies from region to region. Therefore, in the simulation, we integrate all cameras for one density, and in the experiment, each camera produces a density.

**FIGURE 6:**
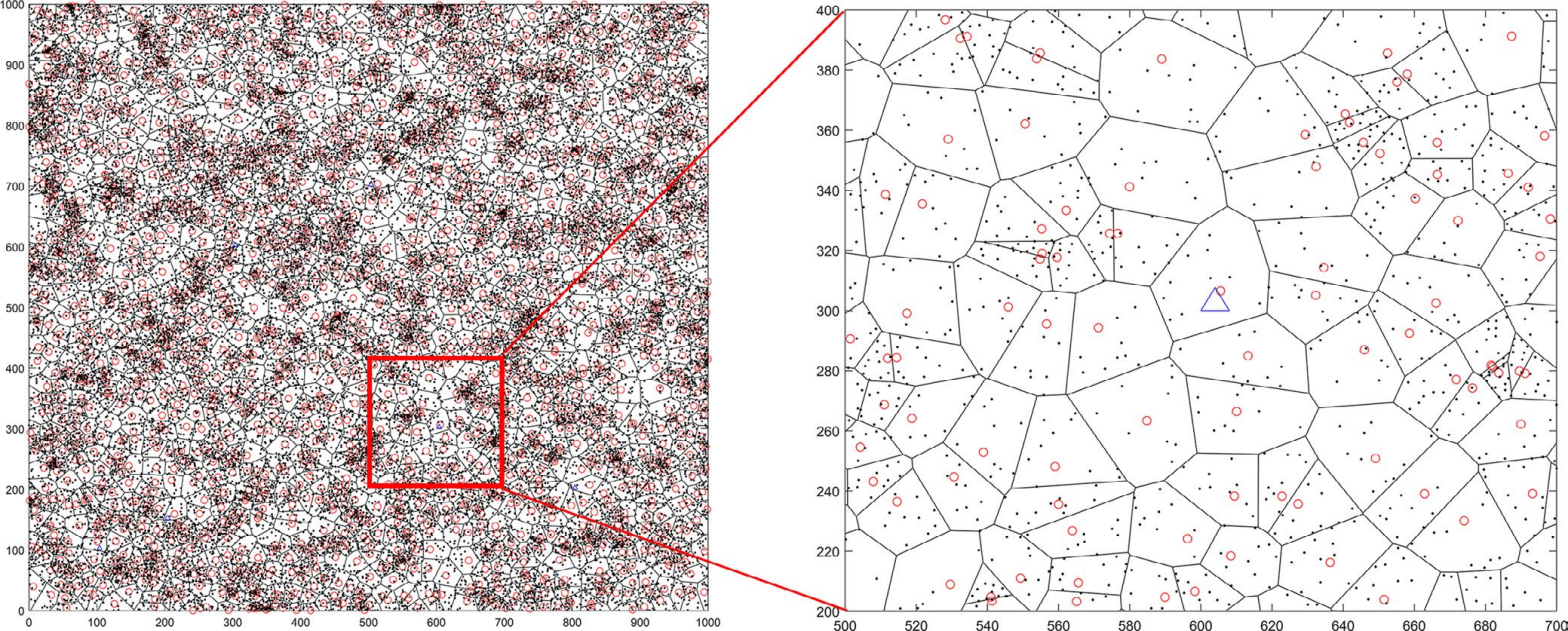
Burrow system (unit: m)

A pika burrow system generated by the algorithm is shown in Figure 6. These points are used to construct a Delaunay triangle network to further divide the Thiessen polygon. Thus, the number of red points equals to the number of burrow systems. Each polygon composed of black solid lines is a Theissen polygon, representing the area occupied by the corresponding burrow system. The black solid dots represent the position of each burrow mouth of the burrow system. The isosceles triangle formed by the blue solid lines indicates the camera's shooting range.

**FIGURE 7:**
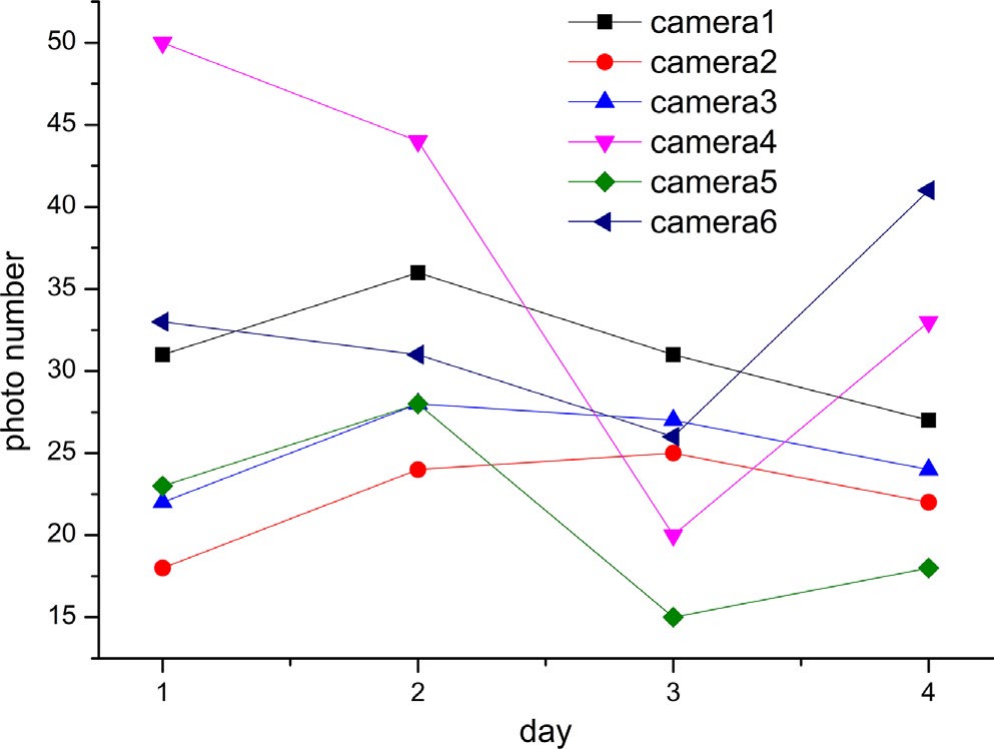
Simulated capture number of the six cameras at different location on different days

Figure 7 shows the simulation results of the capture number of the six cameras in different locations with the given initial value (as shown in Table 3). It can be seen from the figure that the number of shots per day is between 15–50, which varies with the number of days and the position of the camera. The number of shots reflects both the population density and activity intensity of pikas.

**FIGURE 8:**
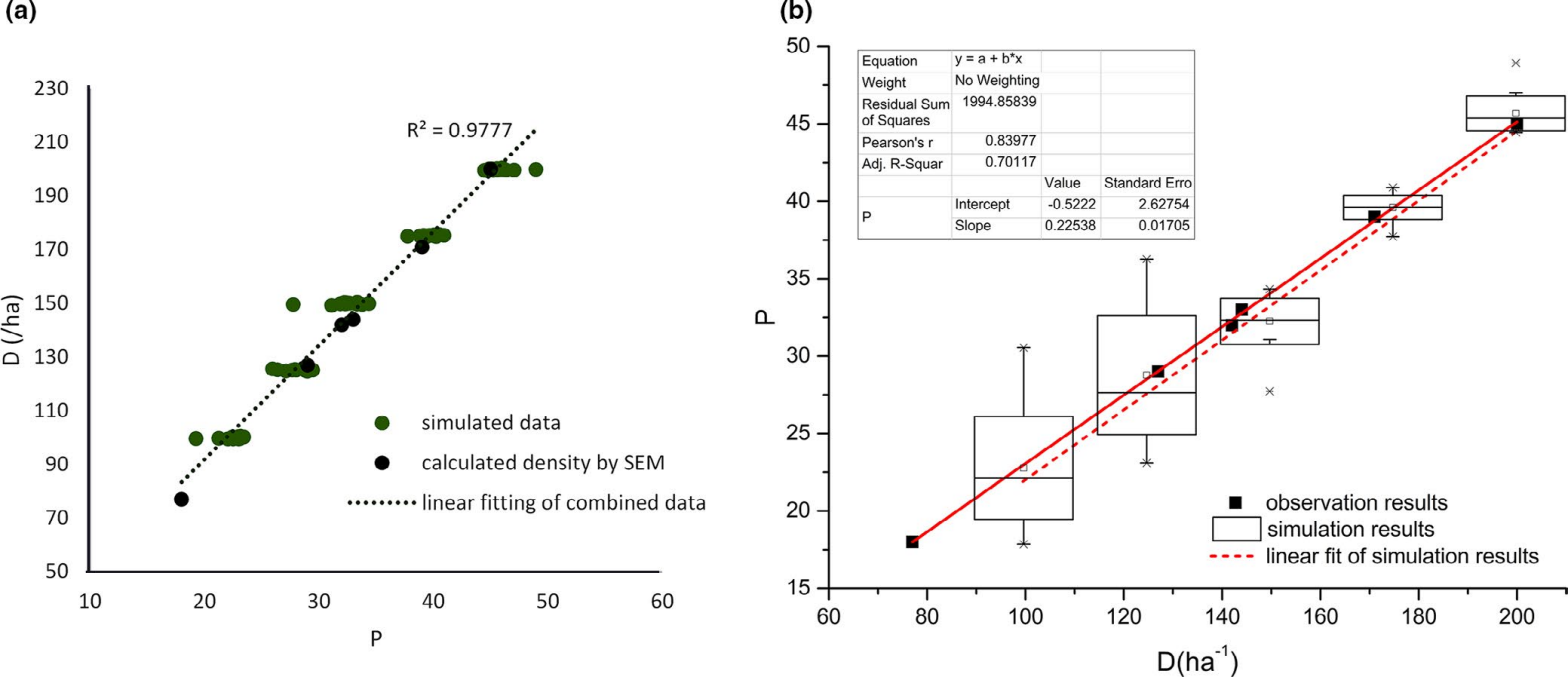
Relationship between the shot frequency *P* and the population density *D* with $v_{\text{large}}=20\;m/\text{min},\;v_{\text{small}}=2\;m/\min$, and $t_{\text{tol}}=15\;\min$

The parameter combination used to derive the similar shot frequencies in Table 3 is as follows: $v_{\text{large}}=20\;m/\min,v_{\text{small}}=2\;m/\text{min}$, and $t_{\text{tol}}=15\;\min$. Different shot frequencies was obtained by changing parameters $Q_{\min}$ and $Q_{\max}$, as shown in Figure 8. It is obvious from Figure 8a that at a given density, the number of shots taken by the camera is concentrated within a relatively small range. Two sets of points show significant similar trends, with $R^{2}=0.98$ using linear fitting. The simulated population density is close to that derived from Equation (1) using observation data giving an approximate counted capture number. In addition, the simulated relationship between shot frequency *P* and population density *D* is also similar to that derived from observations. To further illustrate the relationship, Figure 8a is reversed, so that the slope in Figure 8b represents *λ*. The slop of the linear‐fitting curve of the simulated results in Figure 8b is 0.225, which almost equals the value of 0.226 calculated by Equation (2). Therefore, the model established in this study is capable of representing the behavior of pikas.

This slope is influenced by the pika activity intensity (v) and camera‐shooting range (r,θ). The analysis above shows that the model proposed in this study reflects the quantitative relationship between population density and the shot frequency.

**FIGURE 9:**
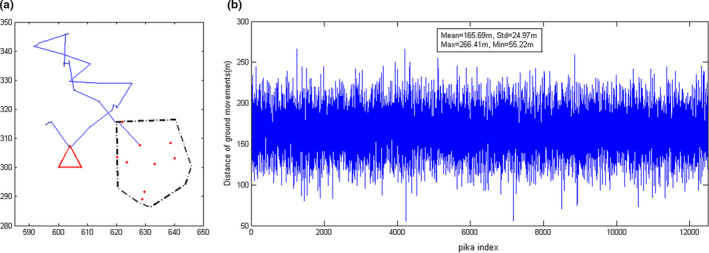
Simulated results given $v_{\text{large}}=20\;m/\min,\;v_{\text{small}}=2\;m/\min$, and $t_{\text{tol}}=15\;\min$: (a) the moving path of a certain pika on a certain day; (b) the moving distance of each pika on a single day

Previous studies have shown that the time range of pikas' ground activity during the peak period is about 600~800s, that is, approximately 10–13.3 min (Song et al., 2015), which approaches the parameters set in the simulation model. In other periods, studies have indicated that within 10 min, the moving distance of a pika is less than 3 m, and the moving distance within a unit step is less than 0.15 m, which is almost neglectable (Wei et al., 2004). Therefore, the peak period mainly reflects the movement behavior of pikas over large distances. Other studies have stated that the daily moving distance of small mammals is approximately 100–300 m (Zhang et al., 2013). To further confirm that the algorithm can reflect the real overview of the pikas’ movement, the distance and path of the pika's activity on a single day is tracked in this study, as shown in Figure 9. Figure 9(a) shows the activity path of a pika in a single day, in which the area enclosed by the solid red line represents the camera‐shooting area, the area enclosed by the dotted‐dash line is the range of the burrow system, the red dots are the burrow mouths, and the blue solid line represents the pika's moving path. Although this roadmap does not reflect the actual movement of each pika, the statistical behavioral information used to describe the entire population is reasonable. Figure 9(b) shows the total distance of each pika's path in a single day, which is in the range of 100–250 m with the mean of 166 m. Such result is also consistent with the current knowledge (Zhang et al., 2013).

## 4 RESULTS AND DISCUSSION

In this section, the effects of several important parameters on the performance of the model were investigated.

### 4.1 Influence of Pika number per burrow system on *λ*

**FIGURE 10:**
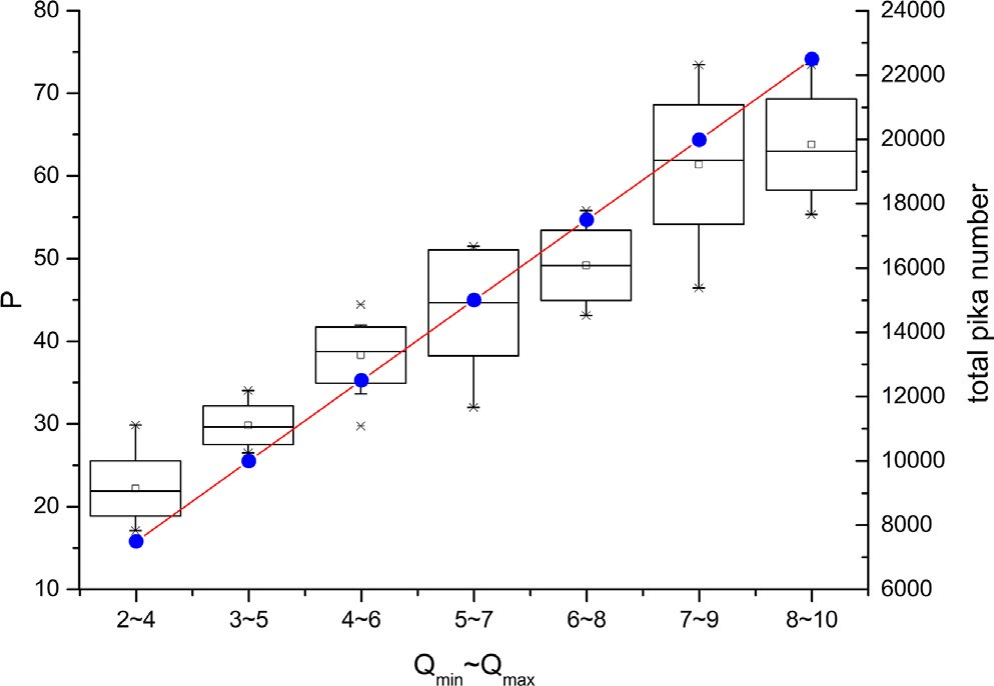
Variation of camera‐shooting frequency with the pika numbers per burrow system, where the box diagram corresponds to the capture number and black solid dots is the total number of pikas (15 trials)

The number of pikas covered in each burrow system directly affects the number of pikas in the entire area, that is, the number of pikas varies with different $Q_{\max}$ and $Q_{\min}$, as does the population density. Figure 10 shows the simulated shot frequency *P* from 15 simulation trials given different numbers of pikas in each burrow system. The total number of pikas in the area shows a linear increasing trend with the increase in the number of pikas in each burrow system. Since the number of burrow systems is sufficiently large in the simulation, the number of pikas generated in each simulation trial approaches $\left(Q_{\max}+Q_{\min}\right)/2*n$. The shot frequency *P* increases with increasing population density. This result is relatively intuitive, as the possibility of shooting pikas in the field also increases when the number of pikas increases.

**FIGURE 11:**
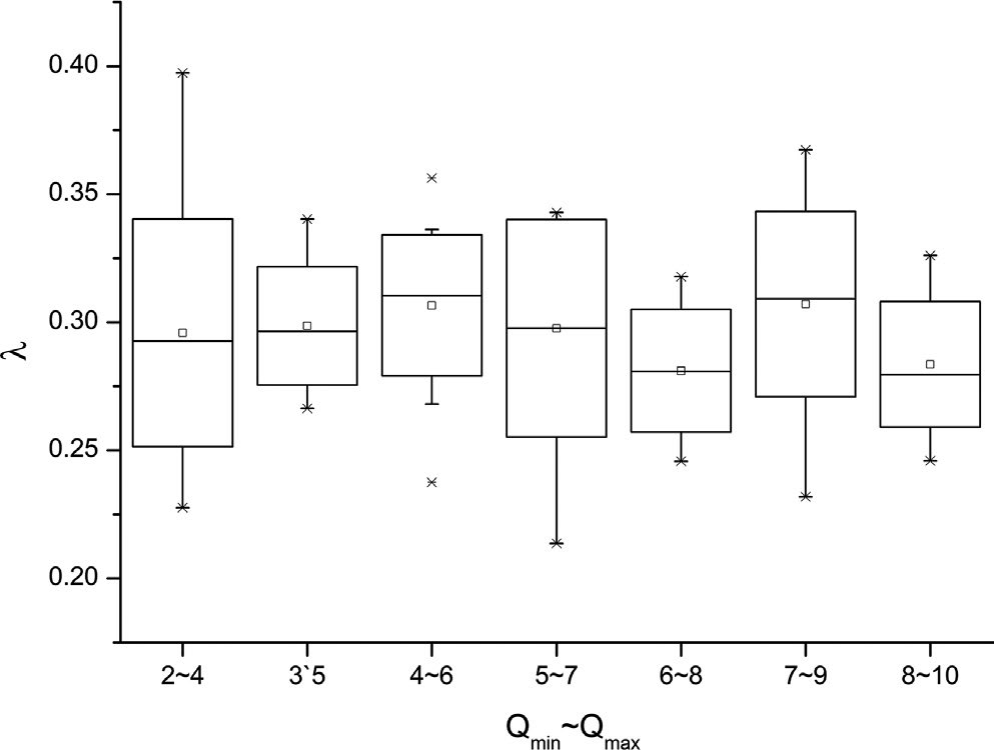
Relationship between the activity intensity of pikas per population density λ and pika number per burrow system (15 trials)

With the given pika activity parameters $t_{\text{tol}}$,$v_{\text{large}}$, and $v_{\text{small}}$, as well as the observation system parameters *r* and *θ*, there is no obvious variation in the calculated pika activity intensity per population density *λ* for different pika number per burrow system, as shown in Figure 11. Using one‐way analysis of variance, no significant difference between the groups in Figure 11 was detected using the significance level of 0.05. The average value of *λ* of different groups falls within a smaller interval of [0.28, 0.3]. According to the definition of *λ* in Equation (2), this result indicates that in a stable behavioral mode, the capture number maintains a stable relationship with the density of pikas. In other words, *λ* does not change with population size with fixed behavioral parameters. This indicates that pika density and capture number would remain a stable proportional relationship regardless of whether these parameters are accurate once they are determined.

### 4.2 Influence of activity time outside burrow on *λ*

**FIGURE 12:**
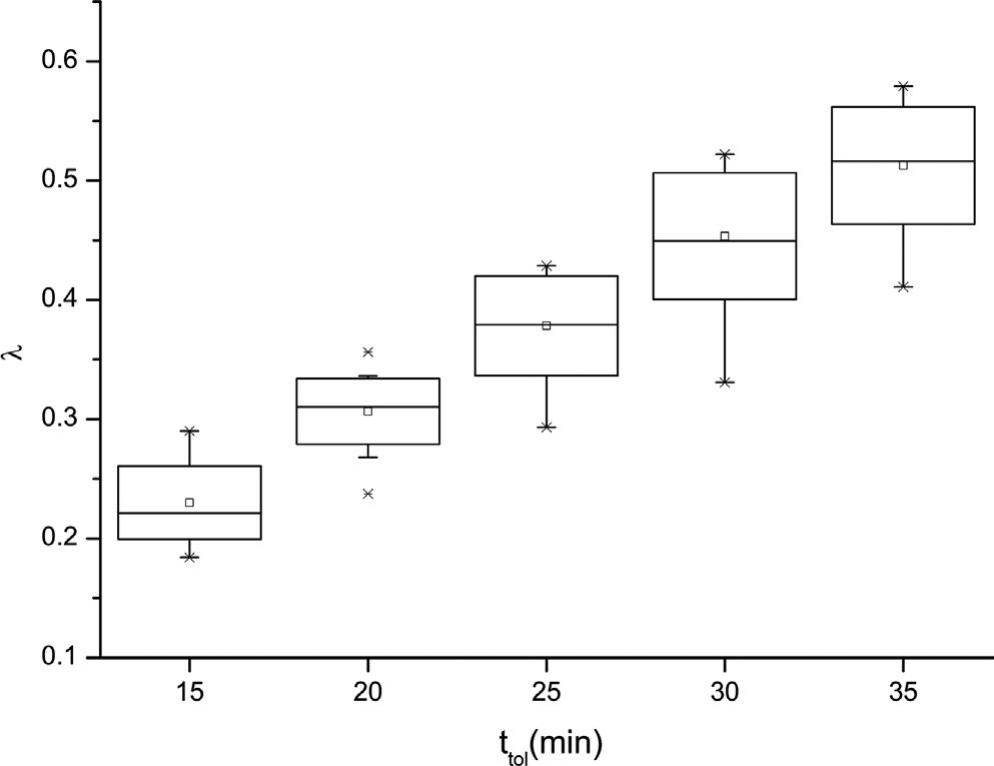
Relationship between activity intensity of pikas per population density λ and pika ground activity time $t_{\text{tol}}$ (15 trials)

Ground activity time refers to the duration the pika stays on the ground, excluding its behavior in the burrow tunnel. Among the activities on the ground, pikas often remain static for a long period of time to be alert to external enemies or to eat and chew. Studies have shown that rest (sitting), vigilance, and eating account for nearly 80% of pikas' ground behavior (Smith et al., 2019). The main purpose of setting the parameter of ground activity time is to reflect the intermittent activity characteristics of the pika and to limit the times and scope of the pika's outing activities. From the results shown in Figure 12, it can be seen that the activity intensity of the pika per population density *λ* and the outing activity time $t_{\text{tol}}$ present an obvious positive correlation. The longer the pika is active on the ground, the more shots are made, and the larger is *λ*.

### 4.3 Influence of activity intensity on *λ*

**FIGURE 13:**
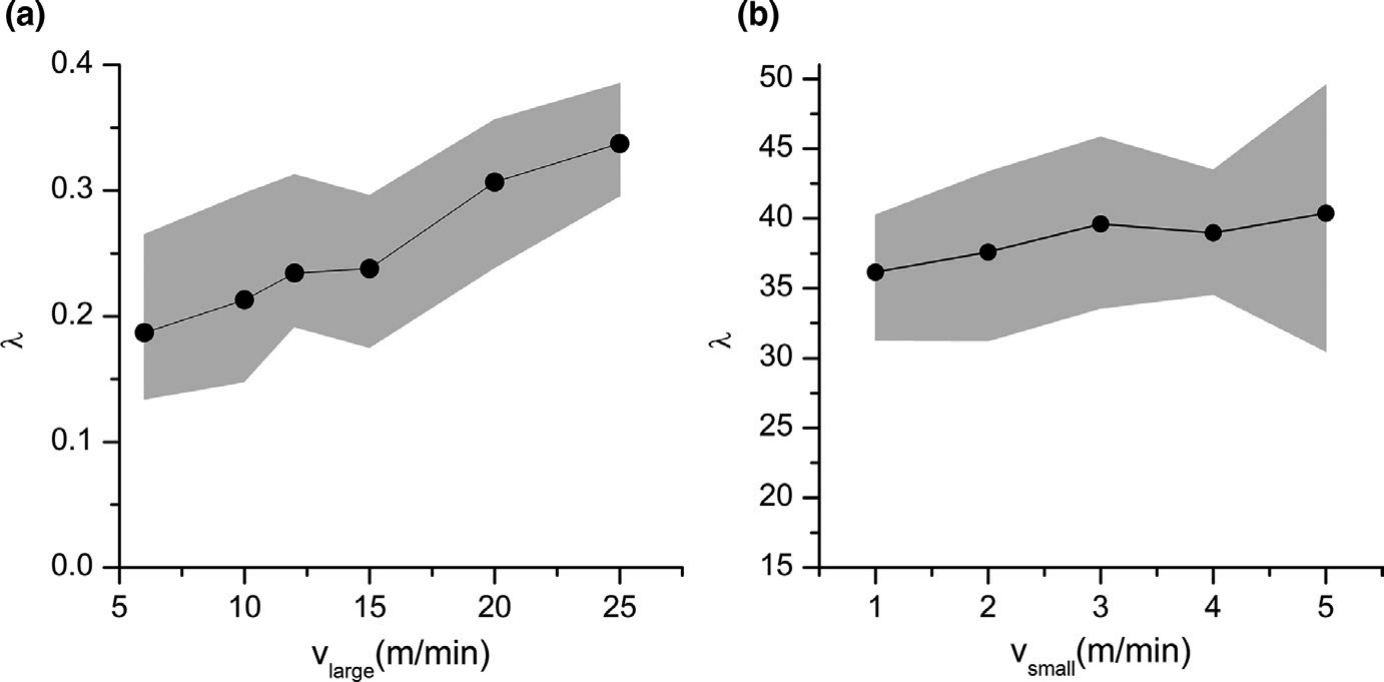
Relationship between activity intensity of pikas per population density λ and pika moving speed of (a) high‐intensity and (b) low‐intensity activity (15 trials)

To investigate the influence of activity intensity on *λ*, different moving speeds of vigorous and low‐intensity activity are set in the simulation model, as shown in Figure 13. The shaded part in Figure 13 represents the variation range of *λ* derived from 15 simulations, and the solid line indicates the mean. Figure 13(a) shows that the rapid movement caused by high‐intensity activity significantly affects the capture number. Although the variation caused by changing the speed of low‐intensity activities is small, there are still significant differences between different groups at the 0.05 significance level using one‐way analysis of variance, that is, $v_{\text{small}}$ also causes the variation in *λ*.

It can be derived from Equation (1) that λ=vr(2+θ)/π. The accumulation of moving displacement at each time step actually reflects the value of v, while *r* and θ are only related to the camera layout, therefore, $v_{\text{large}},\;v_{\text{small}},$ and $t_{\text{tol}}$ have a significant impact on *λ*. These results confirm the consistency between the REM and the simulation model on the basis of Monte Carlo method proposed in this study.

### 4.4 Application and limitation of the method

In addition to proposing an alternative approach to estimate population density based on field observation, this study also provides the characteristic indicators to represent the different behaviors of pikas. The proposed method can be applied to analyze more detailed information compared to existing studies, which there still exists room for improvement.

The rates of pikas’ activity change with environment and climate, which affects density estimation. Based on previous discussion, a relatively stable λ indicates a relative stable behavioral pattern. Such relationship could be investigated further to obtain the response relation between the density of pika and the capture number under changing conditions. Moreover, this model could also be applied to simulate pika activities during peak activity period and nonpeak period separately.

Pang et al. (2020) demonstrated that modest disturbance intensity could increase soil organic carbon and soil total carbon, thus improve the quality of soil and benefit soil carbon sequestration. The “disturbance” in their opinion should be determined by both population distribution and activity intensity, which can be reflected by the parameter λ simultaneously. Indicators including vegetation coverage, ecological diversity, and total biomass can be further discussed using this parameter.

In the observation, the camera is placed only considering the flatness of terrain and the crypticity of the camera. There may be disputes about whether placing cameras closer to the burrow entrances affects pika density estimation. Further research with more cameras is needed to investigate the influence of the camera location on the results. Other practical limitations to camera monitoring such as weather condition and unexpected interference also exist, which can be alleviated by more advanced devices that provide real‐time feedback.

## 5 CONCLUSION

As an important species on the Qinghai‐Tibet Plateau, pikas are of great significance to the ecological environment. In this study, a digital twin model of pikas’ activity composed of a behavior module and a burrow system module is established based on Monte Carlo method. Together with a burrow system divided by Thiessen polygons, the behavioral module is used to simulate the individual behavior of a pika group. An index named activity intensity per population density λ is defined, related to both the pika population density and the shot frequency of the monitoring camera. This index contains information on both the pikas and the monitoring space and thus is applied to validate the simulation model using the shot frequency of the infrared camera in field observations.

With appropriate parameters, the simulation model has good consistency with the REM based on field observations, which is thus applied to study the behavioral characteristics of pikas. The simulation results show that the capture number of the camera is distinctly positively correlated with the number of pikas, which can thus be used as an effective parameter to characterize the density of pikas. The time of outing activities of pikas and the intensity of their behavior are also important parameters affecting the relationship between the number of camera shots and population density. As the ground activity time and exercise intensity increase, the number of shots taken by the camera also increases.

The model essentially establishes the relationship between the pika activity/density parameter and the monitoring space. Given the capture number of a camera, the estimated value of pika density can be derived with a given activity intensity. Moreover, the model is able to simulate the moving path of each pika. Although this simulation cannot be verified for a certain individual, it has statistical significance for a pika population and can be used to study group behavioral characteristics of the pika population. This model can be applied to different scenarios in further studies, such as when the pika's activity changes due to climate change and when a decrease in vegetation causes the pika to spend more time outside, by altering the behavioral parameters.

## CONFLICT OF INTEREST

None declared.

## AUTHOR CONTRIBUTIONS

**Ying‐Hui Jia:** Investigation (equal); Methodology (lead); Software (lead); Visualization (lead); Writing‐original draft (lead). **Fang‐Fang Li:** Conceptualization (equal); Project administration (equal); Supervision (equal); Writing‐review & editing (lead). **Cang Ma:** Data curation (lead); Resources (equal); Validation (supporting). **Guang‐Qian Wang:** Project administration (equal); Resources (equal); Supervision (equal). **Jun Qiu:** Conceptualization (lead); Formal analysis (lead); Funding acquisition (lead); Supervision (lead); Validation (lead).

## ETHICS STATEMENT

The authors declare that they have no conflict of interest. All procedures performed in studies involving human participants were in accordance with the ethical standards of the institutional research committee.

## Data Availability

Sampling locations, code, and original field observation data: Dryad https://doi.org/10.5061/dryad.m905qfv0f.
